# Supplementary material for: Pheromone components affect motivation and induce persistent modulation of associative learning and memory in honey bees
Source: Commun Biol. 2020 Aug 17;3:447. doi: 10.1038/s42003-020-01183-x (PMC7431541; doi:10.1038/s42003-020-01183-x)
Supplement: Supplementary file 1 — Supplementary Information [file 42003_2020_1183_MOESM1_ESM.pdf]

SUPPLEMENTARY INFORMATION

Pheromone components affect motivation and induce persistent modulation of associative learning and memory in honey bees

David Baracchi[1,†,*], Amélie Cabirol[2], Jean-Marc Devaud[1], Albrecht Haase[2,3], Patrizia d'Ettorre[1,4,5‡] & Martin Giurfa[1,5,6,‡,*]

[1] *Research Centre on Animal Cognition, Center for Integrative Biology, CNRS, University of Toulouse, 118 route de Narbonne, F-31062 Toulouse Cedex 09, France*

[2] *Center for Mind/Brain Sciences (CIMeC), University of Trento, piazza Manifattura 1, I-38068 Rovereto, Italy*

[3] *Department of Physics, University of Trento, via Sommarive 14, I-38123 Povo, Italy*

[4] *Laboratory of Experimental and Comparative Ethology, University of Paris 13, F-93430 Sorbonne Paris Cité, France*

[5] *Institut Universitaire de France (IUF), France*

[6] *College of Animal Science (College of Bee Science), Fujian Agriculture and Forestry University, Fuzhou 350002, China*

†Present address: *Department of Biology, University of Florence, Via Madonna del Piano, 6, 50019 Sesto Fiorentino, Italy*

‡ These authors contributed equally to this work.

***Authors for correspondence:** *David Baracchi* (david.baracchi@unifi.it)
Martin Giurfa (martin.giurfa@univ-tlse3.fr)

SUPPLEMENTARY FIGURES

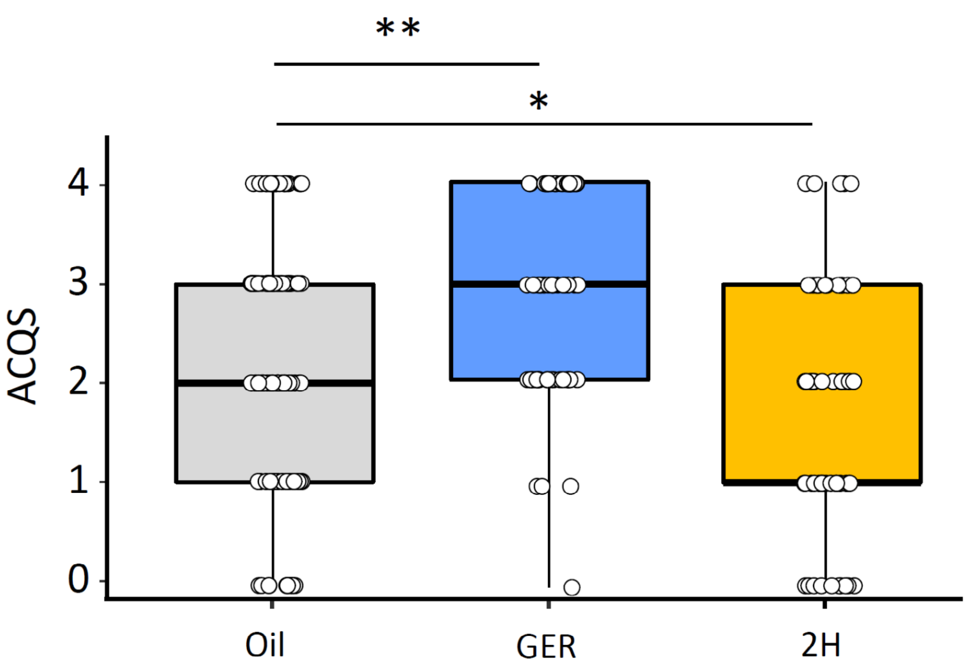

Supplementary Figure 1. Pheromone components modulate individual learning scores according to their valence

Individual learning performance was quantified in terms of an acquisition score (ACQS), which was calculated as the sum of responses of a bee to the five CS+ presentations during associative olfactory PER conditioning. The boxplot with jitter shows the median, quartiles, and max and min (upper and lower whiskers) ACQS values of bees pre-exposed to either GER (n = 75 independent bees), 2H (n = 73 independent bees), or mineral oil (n = 129 independent bees). Individual bees are indicated by the white dots. Pre-exposure to geraniol (GER) and 2-heptanone (2H) induced a significant increase and decrease of ACQS, respectively, with respect to bees exposed to mineral oil. (*) $p = 0.003$; (**) $p = 0.0002$.

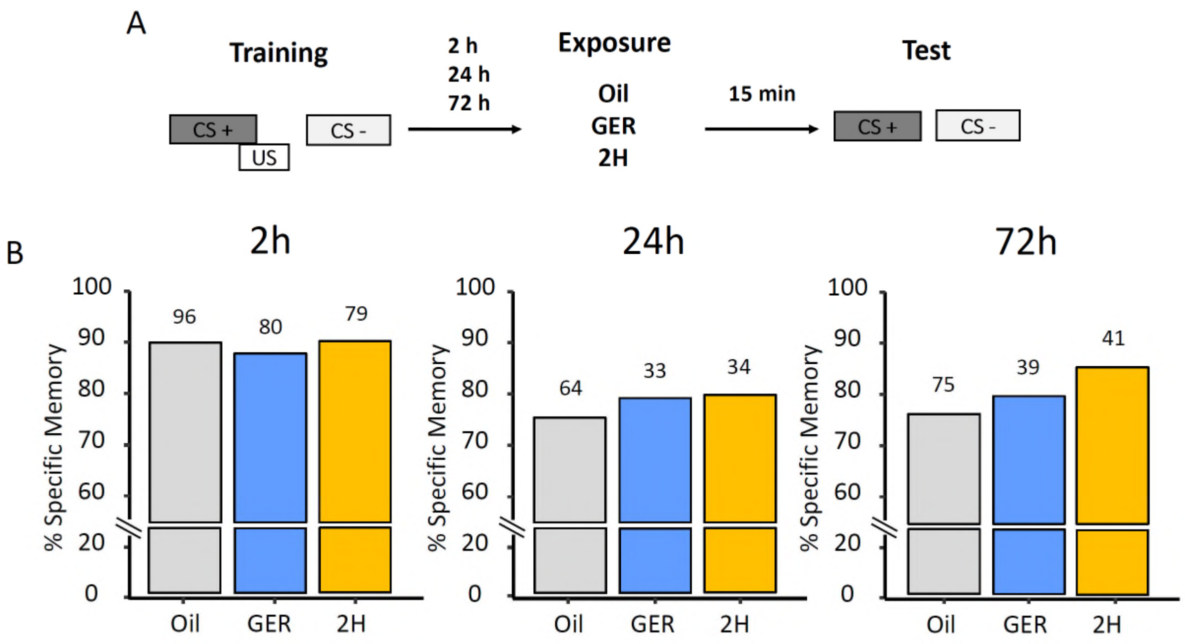

Supplementary Figure 2 A-B. Pre-exposure to pheromone components after conditioning and before memory tests does not affect memory retrieval

A) Experimental protocol used to test the effect of pre-exposure to geraniol (GER) and 2-heptanone (2H) on memory retrieval. Only bees responding correctly in the last conditioning trial (*i.e.* responding to the CS+ and not to the CS-) were pre-exposed and tested. Pre-exposure occurred 15 min before the memory tests performed 2 h, 24 h, or 72 h after conditioning.

B) Proportion of bees showing a specific memory (*i.e.* responding to the CS+ and not to the CS-) when tested for memory retention after conditioning and after being pre-exposed either to GER (n = 80 independent bees), 2H (n = 79 independent bees), or mineral oil (n = 96 independent bees) 15 min before the memory tests performed at 2 h, 24 h, or 72 h. Independent groups of bees were used for each test and pheromone component. Sample sizes are reported above bars. Retrieval performances were similar between groups at any testing time irrespective of exposure. All comparisons were NS.

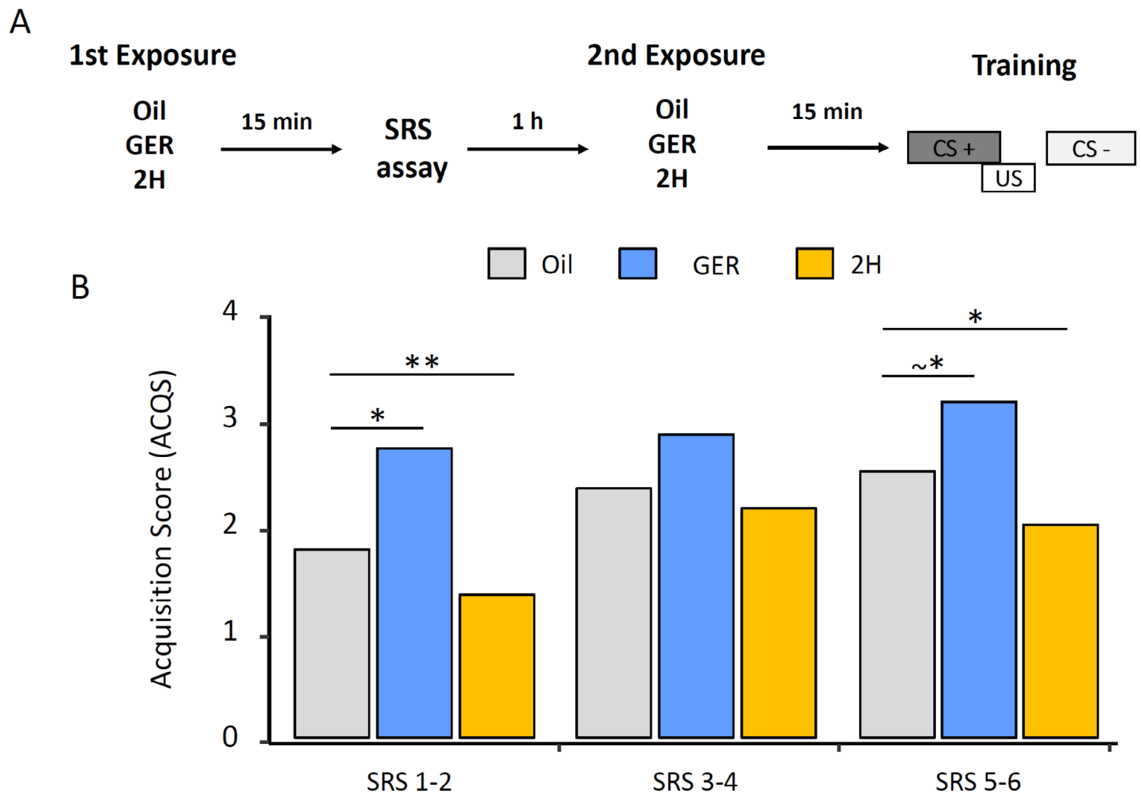

Supplementary Figure 3 A-B. Relationship between sucrose responsiveness (SRS), learning success (ACQS), and pheromone-component exposure.

A) Experimental protocol used. Bees from three categories of sucrose responsiveness (SRS) were segregated according to their learning performance (ACQS) following conditioning.

B) Bees were classified as having low (SRS 1-2), intermediate (SRS 3-4), or high sucrose responsiveness scores (SRS 5-6). Their acquisition score (ACQS) (see Figure S1) is represented according to their pre-exposure treatment. Overall, bees showed an increase of ACQS with SRS (Kruskal-Wallis test, $p < 0.001$), but bees with low (1-2) and high (5-6) SRS were more sensitive to the modulation of learning induced by geraniol (GER) and 2-heptanone (2H) than those with intermediate SRS (Kruskal-Wallis test, SRS_{1-2}: $p < 0.009$, SRS_{3-4}: $p = 0.17$, SRS_{5-6}: $p = 0.05$). GER exposure enhanced ACQS in low SRS bees ($p = 0.047$) and had a marginal non-significant effect ($p = 0.09$) in high SRS bees. 2H induced a non-significant decrease of ACQS with respect to control bees ($p > 0.2$). (*) $p = 0.05$; (**) $p \leq 0.009$.

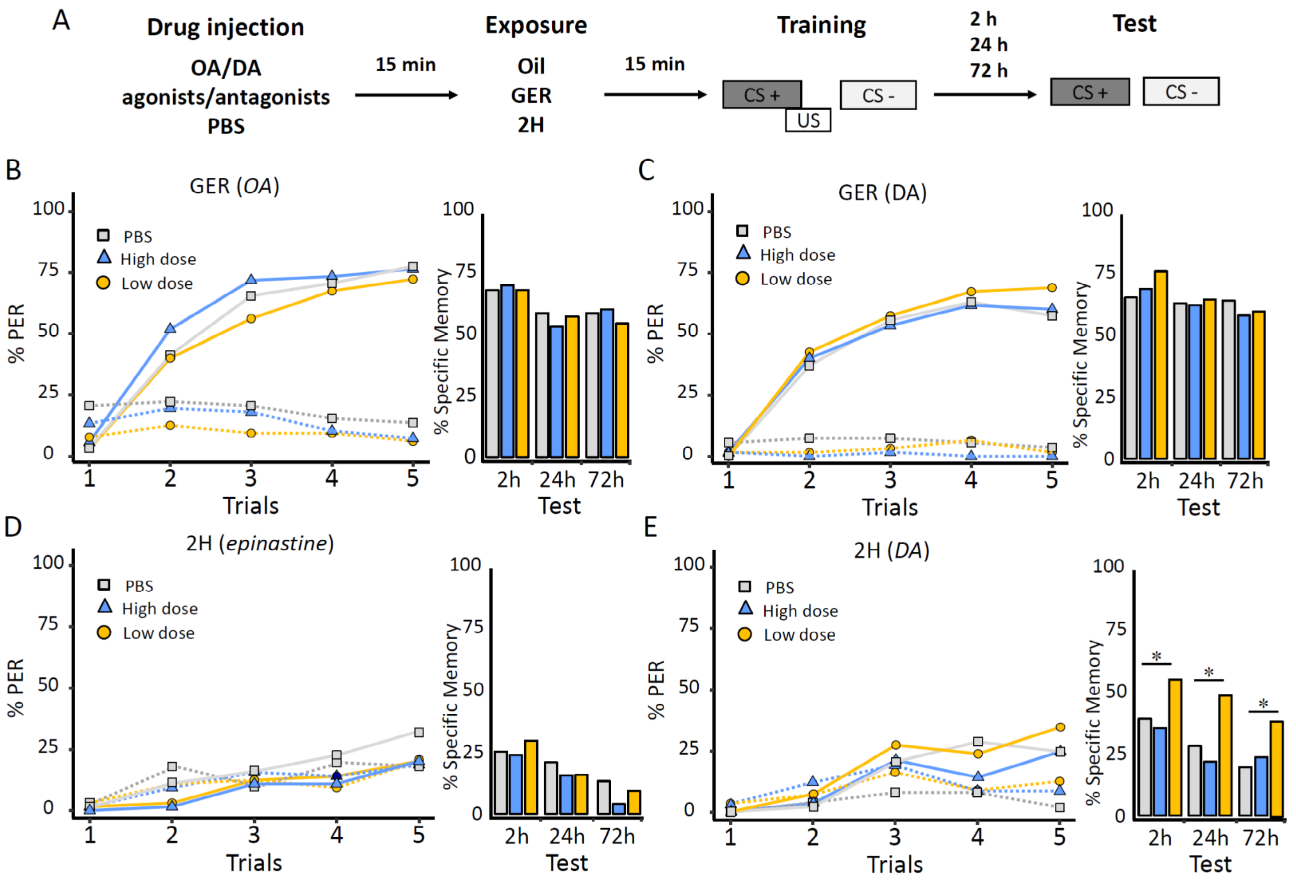

Supplementary Figure 4 A-E. Pharmacological treatment with agonists/antagonists of the dopaminergic and octopaminergic system of honeybees exposed to either GER or 2H.

A) Experimental protocol used.

B) Learning and memory retention of bees injected either with octopamine (OA) [20µM (n = 62 independent bees) or 2mM (n = 65 independent bees)] or PBS (n = 58 independent bees) and pre-exposed to geraniol (GER). Groups were conditioned, and tested in parallel. Learning performance is represented in terms of the proportion of conditioned responses (PER) to the rewarded (full lines) and non-rewarded odors (dotted lines) during five CS+ and CS- trials. Memory retention is represented in terms of the proportion of bees with specific memory *(i.e.* the proportion of bees responding to the CS+ and not to the CS-) in tests performed 2 h, 24 h, or 72 h after conditioning.

C) Same as in B) but for bees injected either with dopamine (DA) [20µM ($n = 61$ independent bees) and 2mM ($n = 60$ independent bees)] or PBS ($n = 54$ independent bees) and pre-exposed to geraniol (GER). Groups were conditioned, and tested in parallel.

D) Bees injected either with epinastine (OA-receptor antagonist) [0.4µM ($n = 53$ independent bees), 4mM ($n = 53$ independent bees)] or PBS ($n = 58$ independent bees) and pre-exposed to 2-heptanone (2H). Groups were conditioned, and tested in parallel.

E) Bees injected either with dopamine (DA) [20µM ($n = 55$ independent bees) and 2mM ($n = 57$ independent bees)] or PBS ($n = 49$ independent bees) and exposed to 2-heptanone (2H). Groups were conditioned, and tested in parallel. (*) $p < 0.05$.

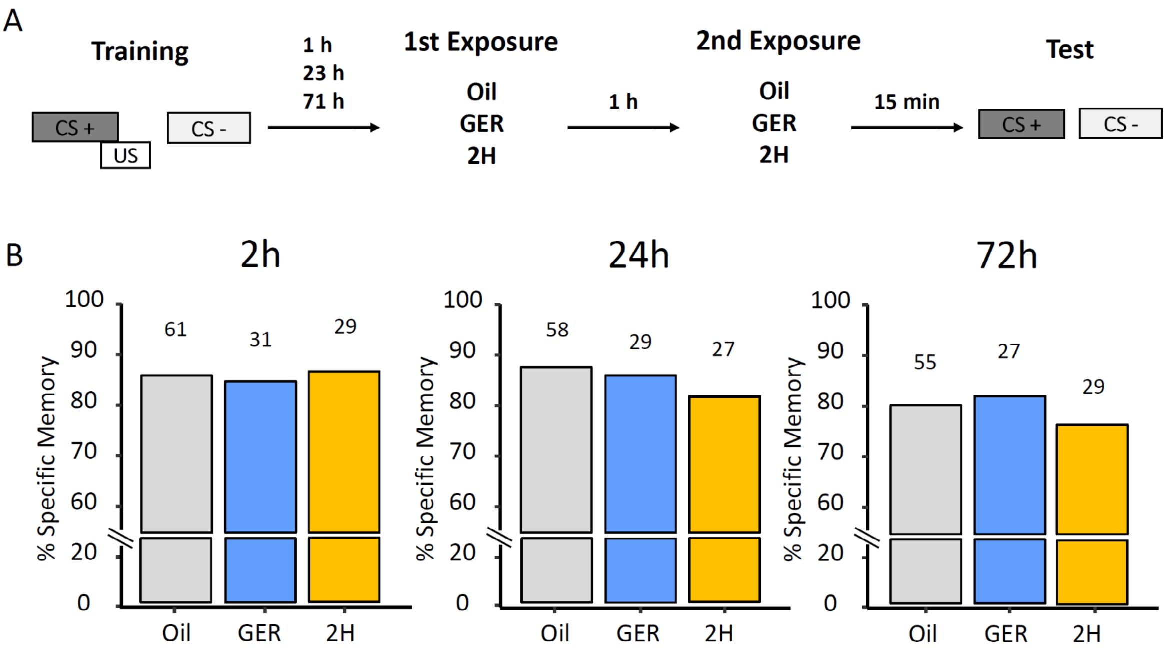

Supplementary Fig. 5 A-B. Pheromone components do not affect memory retrieval when bees are exposed twice after training

A) Experimental protocol used. The experiment was performed to control for the potential difference between bees tested for memory retention after conditioning (Fig. 1B) and bees tested for memory retrieval (Fig. S2) as this comparison showed that pre-exposure to geraniol (GER) and 2-heptanone (2H) affected memory retention (Fig. 1B) but not memory retrieval (Fig. S2). As bees in Fig. 1B were pre-exposed twice to mineral oil/GER/2H (first before a prior assay of sucrose responsiveness, shown in Fig. 3, and second before conditioning, shown in Fig. 1A), while bees in Fig. S2 were pre-exposed once to mineral oil/ GER/2H (after conditioning and before the memory tests), we repeated the memory-retrieval experiment, pre-exposing bees twice after conditioning and before the memory tests. The timing of the pre-exposures corresponded to that experienced by bees in Fig. 1. Only bees responding correctly in the last conditioning trial (*i.e.* responding to the CS+ and not to the CS-) were pre-exposed and tested.

B) Proportion of bees showing specific memory (*i.e.* responding to the CS+ and not to the CS-) when tested for memory retention after being exposed either to GER, 2H, or mineral oil during 15 min prior to the memory tests performed at 2 h, 24 h or 72 h. Independent groups of bees were used for each test and pheromone component. Sample sizes are reported above bars. Retrieval

performances were similar between groups at any testing time irrespective of exposure (Logistic Regression, 2h: *treatment:* $\chi^2 = 0.066$, df: 2, $p = 0.96$, 24h: *treatment:* $\chi^2 = 0.61$, df: 2, $p = 0.74$, 72h: *treatment:* $\chi^2 = 0.3$, df: 2, $p = 0.86$). Thus, pheromone components do not affect memory retrieval, irrespective of the number of pre-exposures.

SUUPLEMENTARY TABLES

Pheromone component	OA		DA		Epinastine		Flupentixol	
	Learning	Memory	Learning	Memory	Learning	Memory	Learning	Memory
GER	=	=	=	=	-	-	-	-
2H	+	+	=	+	=	=	-	+

Supplementary Table 1. Effects induced by pharmacological treatments in honeybees exposed to either geraniol (GER) or 2-heptanone (2H)

The effect of octopamine (OA), dopamine (DA) and their receptor antagonists epinastine (OA) and flupentixol (DA), respectively, on learning and memory of an appetitive olfactory discrimination in honeybees. Drugs were injected into the bee brain upon GER or 2H exposure prior to conditioning and subsequent memory tests. =: no effect; +: increase; -: decrease.
