# Supplementary material for: Pheromone components affect motivation and induce persistent modulation of associative learning and memory in honey bees
Source: Commun Biol. 2020 Aug 17;3:447. doi: 10.1038/s42003-020-01183-x (PMC7431541; doi:10.1038/s42003-020-01183-x)
Supplement: Supplementary file 2 — Reporting Summary [file 42003_2020_1183_MOESM2_ESM.pdf]

nature research

Corresponding author(s): Martin GIURFA (COMMSBIO-20-0635B)

Last updated by author(s): May 15, 2020

Reporting Summary

Nature Research wishes to improve the reproducibility of the work that we publish. This form provides structure for consistency and transparency in reporting. For further information on Nature Research policies, see our Editorial Policies and the Editorial Policy Checklist.

Statistics

For all statistical analyses, confirm that the following items are present in the figure legend, table legend, main text, or Methods section.

n/a	Confirmed	
☐	☒	The exact sample size (*n*) for each experimental group/condition, given as a discrete number and unit of measurement
☐	☒	A statement on whether measurements were taken from distinct samples or whether the same sample was measured repeatedly
☐	☒	The statistical test(s) used AND whether they are one- or two-sided<br>*Only common tests should be described solely by name; describe more complex techniques in the Methods section.*
☐	☒	A description of all covariates tested
☐	☒	A description of any assumptions or corrections, such as tests of normality and adjustment for multiple comparisons
☐	☒	A full description of the statistical parameters including central tendency (e.g. means) or other basic estimates (e.g. regression coefficient) AND variation (e.g. standard deviation) or associated estimates of uncertainty (e.g. confidence intervals)
☐	☒	For null hypothesis testing, the test statistic (e.g. *F*, *t*, *r*) with confidence intervals, effect sizes, degrees of freedom and *P* value noted<br>*Give P values as exact values whenever suitable.*
☒	☐	For Bayesian analysis, information on the choice of priors and Markov chain Monte Carlo settings
☒	☐	For hierarchical and complex designs, identification of the appropriate level for tests and full reporting of outcomes
☒	☐	Estimates of effect sizes (e.g. Cohen's *d*, Pearson's *r*), indicating how they were calculated

Our web collection on statistics for biologists contains articles on many of the points above.

Software and code

Policy information about availability of computer code

Data collection	No software was used for bahavioural data collection<br>LABVIEW 2016 http://www.ni.com/en-us/shop/labview.html was used to collect calcium imaging data
Data analysis	R 3.4.2. https://www.r-project.org/<br>MATLAB R2019A https://nl.mathworks.com/products/matlab.html<br>This study did not generate any new code.

For manuscripts utilizing custom algorithms or software that are central to the research but not yet described in published literature, software must be made available to editors and reviewers. We strongly encourage code deposition in a community repository (e.g. GitHub). See the Nature Research guidelines for submitting code & software for further information.

Data

Policy information about availability of data

All manuscripts must include a data availability statement. This statement should provide the following information, where applicable:

- Accession codes, unique identifiers, or web links for publicly available datasets
- A list of figures that have associated raw data
- A description of any restrictions on data availability

The datasets generated during this study are available at datadryad.org with the following accession ID: https://doi.org/10.6084/m9.figshare.12029526.v1
This study did not generate any new code.

Field-specific reporting

Please select the one below that is the best fit for your research. If you are not sure, read the appropriate sections before making your selection.

☒ Life sciences ☐ Behavioural & social sciences ☐ Ecological, evolutionary & environmental sciences

For a reference copy of the document with all sections, see nature.com/documents/nr-reporting-summary-flat.pdf

Life sciences study design

All studies must disclose on these points even when the disclosure is negative.

Sample size	No statistical methods were used to predetermine exact sample size. Sample sizes were chosen depending on previous research.
Data exclusions	In the sucrose responsiveness assay, bees that did not respond to any sucrose concentration, that responded to water, or that exhibited inconsistent responses to sucrose (i.e. responding to a lower but not to higher sucrose concentrations) were discarded. This is a standard and well-established procedure (Page et al. 1998 J Comp Physiol A 182: 489-500; Scheiner et al. 2001 Neurobiol Learn Mem 2001 76:138-50; Scheiner et al. 2001 Brain Behav Res 120: 67-73; Scheiner et al. 2003, Apidologie, 34(1): 67-72; Scheiner et al. 2004 Apidologie 35: 133-142).<br><br>In all memory tests, bees that did respond neither to the CS- nor to the CS+ during the tests were stimulated with sucrose to check the integrity of the unconditioned response. Bees not responding to sucrose at the end of the retention tests were discarded. This is a standard procedure (Matsumoto, Y., Menzel, R., Sandoz, J. C. & Giurfa, M. J Neurosci Methods 211, 159-167 (2012).<br><br>In the pharmacological experiments, bees losing haemolymph after surgery or not showing drug penetration after a couple of minutes were discarded and never tested.
Replication	We did not perform any replication of within the study. However at least one experiment on sucrose responsiveness successfully confirmed published findings (Baracchi et al Sci Rep. 2017 (1):9875. doi: 10.1038/s41598-017-10113-7)
Randomization	In all experiments, limonene and eugenol were used as conditioned odorants (CSs). Both odors were used either as CS+ or CS- in a counterbalanced design. In olfactory conditioning experiments, odor presentations were pseudo-randomized. Moreover, in all memory tests, the order of presentation of the CS+ and CS- was randomized between bees. All these randomization measures are standard procedures (Bitterman, M. E., Menzel, R., Fietz, A. & Schäfer, S. Classical conditioning of proboscis extension in honeybees (Apis mellifera). J Comp Psychol 97, 107-119 (1983))
Blinding	Blinding was not possible in this study because a single operator performed experiments , which involved many consecutive phases and manipulations, including the exposure of the subjects (honeybees) to odourants and pheromones easily recognizable by the operator. However, blinding was not necessary given that the behavioral readout used throughout was the PER (1 or 0) and therefore unaffected by possible bias.

Reporting for specific materials, systems and methods

We require information from authors about some types of materials, experimental systems and methods used in many studies. Here, indicate whether each material, system or method listed is relevant to your study. If you are not sure if a list item applies to your research, read the appropriate section before selecting a response.

Materials & experimental systems

n/a	Involved in the study	
☒	☐	Antibodies
☒	☐	Eukaryotic cell lines
☒	☐	Palaeontology and archaeology
☐	☒	Animals and other organisms
☒	☐	Human research participants
☒	☐	Clinical data
☒	☐	Dual use research of concern

Methods

n/a	Involved in the study	
☒	☐	ChIP-seq
☒	☐	Flow cytometry
☒	☐	MRI-based neuroimaging

Animals and other organisms

Policy information about studies involving animals; ARRIVE guidelines recommended for reporting animal research

Laboratory animals	Honeybee workers (Apis mellifera) were reared in outdoor hives. In all cases, honey bee foragers (2 to 3-week old) were used.
Wild animals	This study did not involve wild animals

Field-collected samples	Bee foragers were collected each day at a feeder and immediately brought to the laboratory where they were restrained in small tubes, fed and were kept resting in a dark and humid place (ca. 60 %) at 25 ± 1 °C until the start of the experiments (from 1 to 3 days depending on experiments).
Ethics oversight	* Council directive 86/609/EEC defines 'animal' as any live non-human vertebrate – therefore this does not apply to experimental work on insects.

Note that full information on the approval of the study protocol must also be provided in the manuscript.
